# Effects of Surface Charge of Inhaled Liposomes on Drug Efficacy and Biocompatibility

**DOI:** 10.3390/pharmaceutics17030329

**Published:** 2025-03-03

**Authors:** Jinniu Zhang, Yun Huang, Wenhao Shen, Yixing Zeng, Yingjing Miao, Nianping Feng, Tianyuan Ci

**Affiliations:** School of Pharmacy, Shanghai University of Traditional Chinese Medicine, Shanghai 201203, China; zjn2119@126.com (J.Z.); huangyunyun157@163.com (Y.H.); shenwenhao543@163.com (W.S.); zengyixing_2022@163.com (Y.Z.); sean8717@126.com (Y.M.)

**Keywords:** inhalation, liposome, surface charge, pneumonia, anti-inflammation

## Abstract

**Objectives:** Liposomes are a promising drug carrier for inhaled delivery systems and their physical parameters could influence therapeutic efficacy significantly. This study was designed to answer the specific question of the proper surface charge of liposomes in pulmonary inhalation, as well as to study the synergistic anti-inflammation efficacy between drugs. **Methods:** In this work, a series of drug-loaded liposomes with different surface charges (from negative to positive) were prepared, and several in vitro and in vivo assays, including cytotoxicity, hemolysis assay, mucus penetration and lipopolysaccharide (LPS)-induced pneumonia model test, were adopted to evaluate the anti-inflammation efficacy and biocompatibility of the above liposomes. **Results:** Compared with cationic liposomes, anionic liposomes are capable of better mucus penetration and good biocompatibility (low cytotoxicity, better blood compatibility and mild tissue inflammation), but with poor cellular uptake by immune cells. In specific, even when the liposome surface charge was only +2.6 mV, its cytotoxicity and blood hemolysis reached around 20% and 15%, respectively. Furthermore, there was no significant difference in biocompatibility between anionic liposomes (−25.9 vs. −2.5 mV), but a slightly negative-charged liposome exhibited better cellular uptake. **Conclusions:** Thus, slightly negative-charged liposomes (−1~−3 mV) could be a well inhaled drug carrier considering both efficacy and biocompatibility. In an LPS-induced pneumonia mouse model, the drug-loaded liposomes achieved better anti-inflammatory efficacy compared with free drugs.

## 1. Introduction

Pulmonary inhalation plays a vital role in the treatment of pulmonary diseases [1,2,3,4,5] by delivering drugs directly to the sites of action [6,7,8]. However, due to the complex physiological structure of the lungs [2,9], the therapeutic efficacy of pulmonary inhalation is strongly related to mucociliary clearance [10] and mucus layer penetration [11,12]. In specific, the inhalation preparations usually require fine drug particles [13,14], which are generally considered to be 100–500 nm, for the reason that large particles have difficulty reaching bronchioles and small particles are easy to exhale along with breathing [15,16,17].

Liposomes are one of the most prospective delivery vehicles for inhalation due to their good biocompatibility and controllable particle size [18,19]. For example, amikacin liposome [20] inhalation has been approved by FDA for treatment of mycobacterium avium complex (MAC) lung disease [21]. Compared with free amikacin solutions [20], which are rapidly cleared from lungs with short tissue exposure, inhaled drug-loaded liposomes could reduce systemic side effects of drugs and realize the sustained release of drugs in the lung [19].

Even though some research has adopted liposomes [22] as inhalation delivery vehicles [23,24], little attention has been paid to the critical issue of the particle surface charge. In specific, positively charged nanoparticles will be captured by strongly negatively charged mucins through strong electrostatic adsorption, which may hinder their permeability to the tissue, and negatively charged particles more easily penetrate lung mucus and avoid removal and clearance by the oscillation of mucus cilia [25,26,27]. However, on the other hand, positively charged particles could exhibit better cellular uptake and higher therapeutic effects [28].

**Figure 1:**
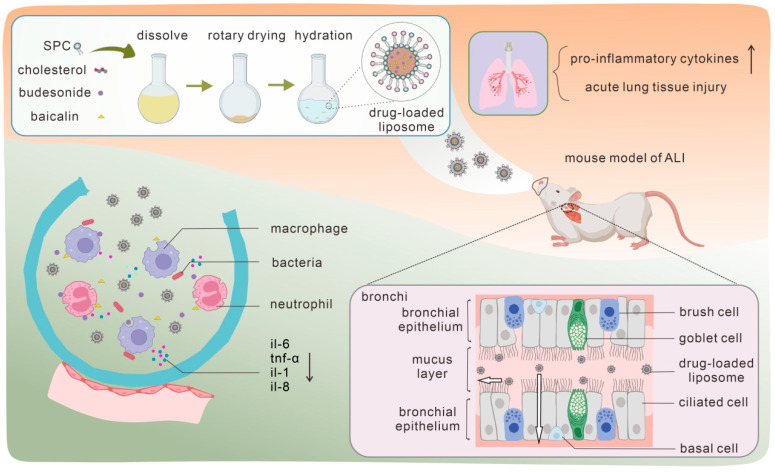
Preparation scheme of the drug-loaded liposomes and their in vivo inhalation anti-inflammation efficacy.

Thus, in this work budesonide (BUD) and baicalin (BAI) co-loaded liposomes with different zeta potentials were designed and prepared to answer this specific question about the proper surface charge of liposomes in pulmonary inhalation, as well as to study the synergistic anti-inflammation efficacy between drugs in a lipopolysaccharide (LPS)-induced pneumonia model (Figure 1).

## 2. Materials and Methods

### 2.1. Materials

Budesonide (BUD), baicalin (BAI), lecithin, cholesterol, and octadecamide were purchased from Aladdin. Distearoyl Phosphatidylglycerole (DSPG) was acquired from Avito (Shanghai) Pharmaceutical Technology Co., Ltd., Shanghai, China. Lipopolysaccharide, DNA (salmon sperm), porcine mucin, and DTPA (ethylene triamine pentaacetic acid) were provided by Shanghai Yuanye Biotechnology Co., Ltd., Shanghai, China. A 50% sterile egg yolk emulsion was purchased from Qingdao Haibo Biological Company. Gelatin, NaCl and KCl were purchased from Shanghai Sinopharm Group Chemical Reagent Co., Ltd., Shanghai, China. Enzyme-linked immunosorbent assay (Elisa) kits (IL-6, TNF-*α*, IL-1*β*) and fluorescent-labeled antibodies (PE-F4/80, APC-CD11b, PE-Ly6G) were purchased from Biolegend Corporation, San Diego, CA, USA. Rhodamine B was supplied by Biyuntian Biotechnology Co., Ltd., Shanghai, China. PBS solution, DMEM cell culture medium, RPMI-1640 cell culture medium, penicillin-streptomycin mixed double antibody, red blood cell lysis buffer and paraformaldehyde fixed solution were purchased from Dalian Meilun Biotechnology Co., Ltd., Dalian, China. Fetal bovine serum was purchased from Ecoxay Bio Ltd., Nanjing, China.

### 2.2. Cell Culture and Animals

Mouse mononuclear macrophage cell lines J774A.1 and RAW264.7 were bought from Shanghai Fuheng Biotechnology Co., Ltd. (Shanghai, China), and cultured with DMEM cell culture medium containing 10% FBS and 1% penicillin/streptomycin in a 37 °C, 5% CO_2_ incubator at saturated humidity for routine culture. The cells were allowed to grow adherent to the wall, and then passaged when they reached 70–80%; cells in the logarithmic growth phase were selected for relevant experiments.

Murine dendritic cell 2.4 line DC2.4 was bought from Shanghai Biological Technology Co., Ltd. (Shanghai, China) and cultured in RPMI-1640 cell culture medium with 10% FBS and 1% penicillin/streptomycin in a 37 °C, 5% CO_2_ incubator at saturated humidity for routine culture. The cells were allowed to grow adherent to the wall, and then passaged when they reached 70–80%; cells in the logarithmic growth phase were selected for relevant experiments.

Balb/c female mice, weighing 18–22 g, were purchased from Weitong Lihua Laboratory Animal Technology Co. Ltd. Beijing, China and raised in the Animal Laboratory Center of Shanghai University of Traditional Chinese Medicine with a relative humidity of 40–60% and room temperature of 20–25 °C. The mice were fed and drank freely during the experiment, and were accommodated for 1 week before the experiment. All animal experiments were conducted in accordance with the relevant regulations of the Experimental Animal Welfare and Ethics Committee of Shanghai University of Traditional Chinese Medicine (PZSHUTCM2502240002; 23 September 2024).

### 2.3. Determination of the Combination Index (CI) of BUD and BAI

The synergistic anti-infection efficacy of the combination of BUD and BAI was evaluated by the extent of cytokine secretion of J774A.1 cells after LPS stimulation. In brief, the half-maximal inhibitory concentration (IC_50_) of budesonide and baicalin was first determined. J774A.1 cells were inoculated in 24-well plates (1 × 10^5^/well) and stimulated by adding LPS (50 ng/mL) to the medium. After 2 h, either BUD solution with a final medium concentration of 0.5, 1, 5, or 10 μM or BAI solution with a final medium concentration of 1, 5, 20, or 35 μM was added into the cell medium. After further incubation for 6 h, the culture supernatant of each well was collected and centrifuged (3000 rpm, 10 min). IL-6 concentration of the supernatant was determined by ELISA kit, and IC_50_s of BUD (IC_50,BUD_) and BAI (IC_50,BAI_) were calculated by Compusyn software 1.2.

The combination ratios of BUD and BAI were set to be 100% IC_50,BUD_, 95% IC_50,BUD_ + 5% IC_50,BAI_, 85% IC_50,BUD_ + 15% IC_50,BAI_, 60% IC_50,BUD_ + 40% IC_50,BAI_, 50% IC_50,BUD_ + 50% IC_50,BAI_, 40% IC_50,BUD_ + 60% IC_50,BAI_, 15% IC_50,BUD_ + 85% IC_50,BAI_, 5% IC_50,BUD_ + 95% IC_50,BAI_ and 100% IC_50,BAI_. The IL-6 secretion of J774A.1 cells after LPS stimulation were determined with same methods above to determine the combination index of BUD and BAI.

### 2.4. Preparation of BUD and BAI Co-Loaded Liposomes (Drug@Lip)

Amounts of 1.45 mg BUD, 1.45 mg BAI, 26.1 mg soy lecithin, and 6.5 mg cholesterol were placed in a round-bottom bottle and dissolved in a mixed solvent of chloroform and methanol (3:4, *v*/*v*). The organic solvent was removed under vacuum by a rotary evaporator with a rotation speed of 60 rpm to form a homogeneous lipid film. A solution of 6 mL PBS was added to the bottle, and the lipid film was hydrated for 2 h to obtain a pale-yellow lipid suspension. Then, the lipid suspension was sonicated twice for 15 min using a 40 HZ probe in an ice bath, and further passed through 0.45 μm and 0.22 μm filters to obtain liposomes with smaller and more homogeneous particle sizes. This prepared drug-loaded liposome was stored in a 4 °C refrigerator before use, and named Drug@Lip-B.

To prepare liposomes with different zeta potentials, the compositions of the lipids were adjusted. To obtain anionic liposomes, DSPG was added to the formulation. The lipid composition was 1.45 mg BUD, 1.45 mg BAI, 17.4 mg soy lecithin, 6.5 mg cholesterol, and 13 mg DSPG for Drug@Lip-A. To obtain cationic liposomes, octadecylamine was added to the formulation. The lipid composition was 1.45 mg BUD, 1.45 mg BAI, 26.1 mg soy lecithin, 6.5 mg cholesterol, and 0.5 mg octadecylamine for Drug@Lip-C. The lipid composition was 1.45 mg BUD, 1.45 mg BAI, 26.1 mg soy lecithin, 6.5 mg cholesterol, and 6 mg octadecylamine for Drug@Lip-D.

### 2.5. Characterization of Drug-Loaded Liposomes

The particle size, polydispersity index (PDI) and zeta potential were determined by dynamic light scattering (DLS, Nazo ZS 90, UK Malvern Inc., Malvern City, UK). The PDI reflects the degree of non-uniformity of a size distribution of particles and ranges from 0 to 1 [29]. Generally, a smaller PDI indicates that the particle size distribution of liposomes is more concentrated and uniform [30].

The encapsulation efficiency (EE) and drug loading capacity (DL) of each group were also determined. To determine the free drug content in the liposome solution, 600 μL drug-loaded liposome solution was added into the ultrafiltration centrifuge tube (MWCO 30 KDa) and centrifuged at 12,000 rpm for 20 min. The filtrate was sucked from the outer compartment of the centrifuge tube, and the contents of BUD and BAI in the filtrate, which were the unloaded drug, were measured by HPLC. Another 400 μL drug-loaded liposome solution was added to the tube, and 1600 μL acetonitrile was added as well. The mixture was treated by ultrasound (40 HZ, 20 min) and passed through a 0.22 μm needle-type filtration membrane. The contents of BUD and BAI in the solution, which were the total contents of the drug in the liposome solutions, were determined by HPLC. The encapsulation efficiency (EE) and drug loading capacity (DL) were calculated according to the formulas

EE (%) = W_drug loaded_/W_drug added_ × 100

DL (%) = W_drug loaded_/W_total liposome_ × 100

where W_drug loaded_ is the drug encapsulated into the liposome, W_drug added_ is the drug added in the preparation of the liposome, and W_total liposome_ is the mass of the liposome with drug encapsulated.

### 2.6. Stability of Drug-Loaded Liposomes

The liposome solution was stored at 4 °C for 25 days, and the samples were taken out at day 1, day 7, day 14 and day 25. After dilution with PBS, the particle size was measured by DLS.

### 2.7. Cellular Toxicity of Charged Blank Liposomes

CCK-8 assays were performed to assess the cell viability of RAW264.7 and DC2.4 cell lines after incubation with charged blank liposomes of different zeta potential. The cells were inoculated in 96-well plates at a density of 1 × 10^4^ per well. The blank liposomes with different zeta potentials (Lip-A, Lip-B, Lip-C, Lip-D) were added to the medium to achieve final lipid concentrations of 0, 0.5, 1, 2.5 and 5 mg/mL. After incubation for 24 h, the cell culture medium was removed and CCK-8-containing medium was added and incubated for another 4 h. The absorbance value at 492 nm was recorded on a microplate reader (Speotra Max 190, Bio-RAD Corporation, Hercules, CA, USA). The absorbance of the negative control group (lipid concentration of 0 mg/mL) was defined as 100%.

### 2.8. In Vitro Hemolysis of Charged Blank Liposomes

Whole blood was collected from the mice via the orbital vein and centrifuged at 1200 rpm for 10 min. The precipitates (blood cells) were resuspended in saline to make 2% (*v*/*v*) erythrocyte solution. The prepared erythrocyte solution was mixed with equal amounts of blank liposome solutions with final lipid concentrations of 0.5, 1, 2.5 and 5 mg/mL. After incubation at 37 °C for 1 h, the samples were centrifuged at 1500 rpm for 5 min, and the supernatant was taken out to determine the absorbance at 540 nm. PBS was used as the negative control and deionized water was used as the positive control. The haemolysis rate of blood erythrocytes was determined by the formula

Hemolysis (%) = (A_sample_ − A_0%_)/(A_100_ − A_0%_) × 100%

where A_100%_ and A_0%_ are the absorbance values of the positive control and negative control, respectively; A_sample_ is the absorbance value of the experimental group.

### 2.9. In Vitro Artificial Mucus Permeability of Charged Liposomes

The mucus permeation of charged liposomes with different zeta potentials was analyzed using an artificial mucus model. Rhodamine B (RhB) was used as a model drug in this assay and encapsulated into the liposomes, and the red color of RhB helped the observation of the penetration of drug-loaded liposomes into the artificial mucus. The artificial mucus was prepared according to the published literature [31,32]. In brief, 1000 mg of DNA, 500 μL of sterile egg yolk emulsion, 500 mg of porcine gastric mucin, 0.59 mg of DTPA, 500 mg of NaCl, 220 mg of KCl, and 2 mL of RPMI medium were added to 100 mL of deionized water, and the mixture was stirred thoroughly with a glass rod and incubated for 2 h at a constant temperature of 37 °C in an incubator to obtain 50 mL the artificial mucus solution. In the meantime, 10% (*w*/*v*) gelatin solution was prepared and separately added into several 2 mL centrifuge tubes, waiting for it to harden at room temperature. After that, 0.75 mL of artificial mucus was slowly added to the upper layer of gelatin, and then 0.5 mL of the liposome solution was slowly added to the upper layer of each group of artificial mucus. Finally, the centrifuge tubes were incubated in an oven at 37 °C, and at certain time intervals (0.5 h, 2 h, 4 h), the tubes were removed to observe photographs and to record the penetration of each group of liposomes. After the last observation, the top layer of the centrifuge tube was discarded and the gelatin was incubated for 30 min at 37 °C in an oven to liquefy the gelatin, and the absorbance of the gelatin solution at 552 nm (the absorbance wavelength of Rhodamine B) was measured by microplate reader and the value of penetration of liposomes into the mucus layer was calculated and assessed.

### 2.10. Cellular Uptake of Drug@Lip-A and Drug@Lip-B

In order to study the cellular uptake ability of Drug@Lip-A and Drug@Lip-B, Rhodamine B was used as a model drug to assess the cellular uptake of different drug-loaded liposomes, which were named RhB@Lip-A and RhB@Lip-B, respectively.

RAW264.7 cells were cultured in 35 mm confocal dishes at a density of 2 × 10^5^ cells. Then, the cells were incubated with PBS, RhB@Lip-A and RhB@Lip-B for another 4 h, respectively. After that, the cells were washed with PBS and the fluorescence of the cells was observed by confocal laser microscopy (LSM710, ZEISS, Oberkochen, Germany). Meanwhile, the cells were also transferred to flow tubes to compare the fluorescence signals of each group by flow cytometry (CytoFLEX, Beckman, Pasadena, CA, USA).

### 2.11. In Vivo Biocompatibility of the Blank Liposomes After Inhalation

In vivo biocompatibility of blank liposomes with different zeta potentials was investigated. Balb/c female mice were randomly divided into groups, saline, Lip-A, Lip-B, Lip-C and Lip-D. Sample solutions of 100 μL were administered by inhalation once a day for three consecutive days to the mice. After 30 days, the mice were euthanized and the primary organs (heart, liver, spleen, lungs and kidneys) were collected for hematoxylin and eosin (H&E) staining and observation of the microstructure and inflammatory status of each organ.

### 2.12. In Vivo Anti-Inflammatory Efficacy in the LPS-Induced Pneumonia Model

Balb/c mice were anesthetized with isoflurane and 50 μL LPS (10 mg/kg) was instilled into the trachea by endotracheal intubation with a 1 mL syringe needle to set up the pneumonia model.

The experimental groups were set as: saline, BUD (1.25 mg/kg), BUD and BAI (BUD 1.25 mg/kg, BAI 1.85 mg/kg); BUD and BAI co-loaded anionic liposome (Drug@Lip-B. BUD 1.25 mg/kg, BAI 1.85 mg/kg). The drug was administered by inhalation through a commercially used nebulizer (NEB-002, Qingdao Future Mobile Medical Technology Co., Ltd., Qingdao, China).

Post-24 h from drug inhalation, blood was collected from the orbital vein. After centrifugation at 3000 rpm for 10 min, the upper layer of serum was collected and the concentrations of IL-6, TNF-*α* and IL-1*β* were determined by Elisa kits.

After blood collection, the mice were sacrificed and bronchoalveolar lavage fluid (BALF) was collected. In brief, a 22 G catheter was inserted into the trachea and 1 mL cold PBS (containing 100 μM EDTA) was slowly injected into the lungs for lavage. The lavage fluid was recovered within 2 min and the procedure was repeated three times. The supernatant was obtained by 3000 rpm centrifugation for 10 min to evaluate the protein level in BALF via a BCA protein detection kit. The precipitated cells were re-suspended in 200 μL red blood cell lysis buffer for 2 min. An amount of 1 mL PBS was added to stop the lysis and the mixture was centrifuged at 3000 rpm for 7 min. The remaining cells were suspended in PBS (1% FBS) and the percentages of macrophages (F4/80^+^CD11b^+^) and neutrophils (Ly6G^+^CD11b^+^) were analyzed by flow cytometry after antibody staining.

The lung tissues were scissored out for the following assays. The right lung lobe was fixed with 4% paraformaldehyde for 48 h, then dehydrated and embedded in paraffin wax for H&E staining and observation. The left lung lobe was weighed immediately with a high-precision microgram electronic scale after being slightly treated with filter paper to remove the surface blood and recorded as wet weight (W). Then, the lung tissue was dried in a thermostat at 65 °C and was weighed and recorded as dry weight (D). The wet-to-dry ratio of the lung, W/D, was calculated for assessing the level of pulmonary oedema.

### 2.13. Statistical Analysis

The data were expressed as mean ± SD. Significant differences were analyzed with Student’s *t*-test between two groups and one-way ANOVA for three or more groups. The survival curve was analyzed by Mantel–Cox test. All statistical analyses were conducted with GraphPad Prism 9.3 and Compusyn software 1.2. The threshold of a statistically significant difference was defined as *p* < 0.05.

## 3. Results

### 3.1. Synergistic Anti-Inflammation Effects of Budesonide and Baicalin

**Figure 2:**
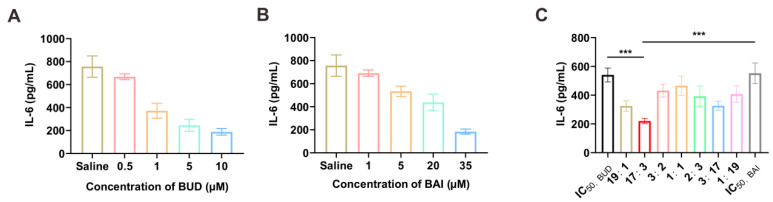
Synergistic anti-inflammation effects of budesonide and baicalin. (**A**,**B**) IL-6 secretion after treatment with BUD (**A**) and BAI (**B**) of J774A.1 cells after stimulation by LPS (*n* = 6). (**C**) IL-6 secretion after treatment with the combination of BUD and BAI (*n* = 6). Ratio 19:1 indicates 95% IC_50,BUD_ + 5% IC_50,BAI_; 17:3 indicates 85% IC_50,BUD_ + 15% IC_50,BAI_; 3:2 indicates 60% IC_50,BUD_ + 40% IC_50,BAI_; 1:1 indicated 50% IC_50,BUD_ + 50% IC_50,BAI_; 2:3 indicates 40% IC_50,BUD_ + 60% IC_50,BAI_; 3:17 indicates 15% IC_50,BUD_ + 85% IC_50,BAI_; 1:19 indicates 5% IC_50,BUD_ + 95% IC_50,BAI_. Statistical significance was calculated via Student’s *t*-test. All data are expressed as mean ± SD, *** *p* < 0.001.

We analyzed the synergistic anti-inflammation effects of budesonide and baicalin by evaluating the cytokine secretion of immune cells after drug treatments. As shown in Figure 2A,B, the half maximum inhibitory concentrations (IC_50_) of BUD and BAI on IL-6 secretion in J774A.1 cells were 2.13 μM and 17.82 μM, respectively, and the anti-inflammation effects were significantly enhanced within a wide combination range from 1:19 to 19:1 compared with BUD or BAI alone (Figure 2C), in which the best combination ratio was 85% IC_50,BUD_ + 15% IC_50,BAI_ (CI value of 0.13) with a mass ratio of 1:1.68 (Table S1).

### 3.2. Characterization of Drug-Loaded Liposomes with Different Surface Charges

Liposomes were prepared by the thin-film hydration method and the particle surface charge was adjusted by changing the lipid composition while preparation. In specific, the addition of octadecylamine could yield positive-charged liposomes, while the addition of DSPG could yield negative-charged liposomes. The particle size, PDI and zeta-potential, drug encapsulation efficacy (EE) and loading capacity (DL) of prepared liposomes were listed in Table 1, and the liposomes were recorded as Drug@Lip-A (negative-charged), Drug@Lip-B (slightly negative-charged), Drug@Lip-C (slightly positive-charged), and Drug@Lip-D (positive-charged), with zeta potentials changing from −25.9 to +21.5 mV. We further evaluated the drug loading capacity of the liposomes, and the mass ratio of BUD and BAI ranging from 1:1 to 1:1.5, which was in the best range for the synergistic combination ratio.

The liposome stability during storage were also analyzed. As shown in Table S2, the change in particle size was within 17.7% (Drug@Lip-A), 15.5% (Drug@Lip-B), 7.8% (Drug@Lip-C), and 28.8% (Drug@Lip-D) after 25 days of storage, indicating good stability of the liposomes.

### 3.3. Biocompatibility of Liposomes with Different Surface Charges

#### 3.3.1. Cytotoxicity

**Figure 3:**
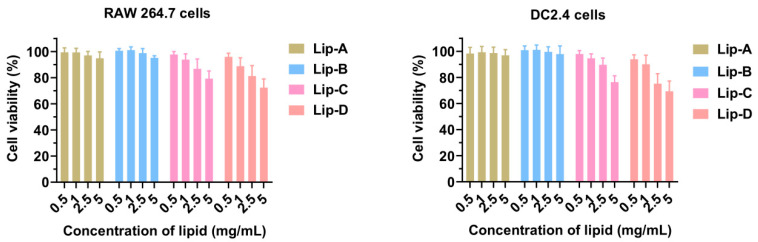
In vitro cytotoxicity of liposomes on RAW264.7 cells (**left**) and DC2.4 cells (**right**) (*n* = 5). Data are expressed as mean ± SD.

The cytotoxicity of blank liposomes with different surface charge was first evaluated by CCK-8 assay with the mononuclear macrophage cell line RAW264.7 and dendric cell line DC2.4 cell as the model cells. After 24 h incubation of blank liposomes (lipid concentration 0, 0.5, 1.0, 2.5, or 5.0 mg/mL), the cell viabilities of different groups were evaluated. As shown in Figure 3, the negative-charged liposomes (Lip-A and Lip-B) were less toxic to cells compared with positive-charged liposomes (Lip-C and Lip-D), with the cell viability being 94.8% (Lip-A), 95.2% (Lip-B), 76.43% (Lip-C), and 69.34% (Lip-D) for RAW264.7 cells, indicating little cytotoxicity for negative and slightly-negative liposomes.

#### 3.3.2. Blood Compatibility

**Figure 4:**
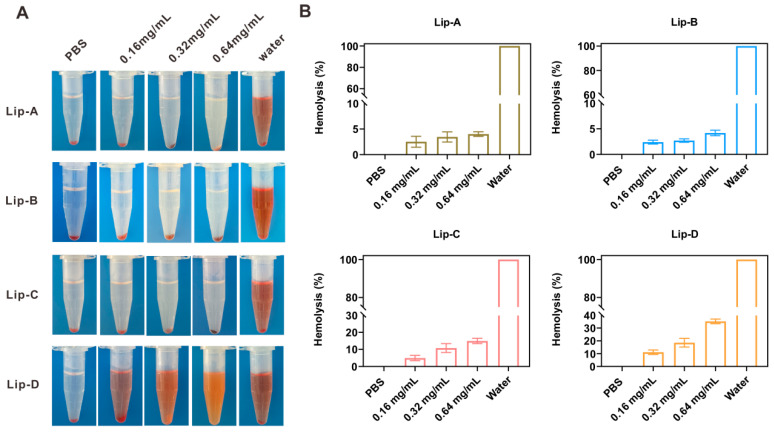
Blood compatibility of blank liposomes with different surface charges. (**A**) Typical images of the hemolysis of red blood cells after centrifugation. (**B**) Hemolysis percentage of the blood cells after incubation with blank liposomes at 37 °C for 1 h (*n* = 4). The hemolysis percentages of positive (distilled water) and negative controls (normal saline) were defined as 100% and 0%, respectively.

The hemolysis of erythrocytes in the liposome solutions was visualized (Figure 4A), and the hemolysis percentages were 4.0% (Lip-A), 4.2% (Lip-B), 15.0% (Lip-C) and 35.2% (Lip-D), respectively, when the lipid concentration reached 0.64 mg/mL (Figure 4B). Generally, the material could be considered of good blood compatibility if the erythrocyte hemolysis is less than 5%; therefore, the positive-charged liposomes, even if the zeta-potential was only 2.6 mV, were of poor blood compatibility and could cause hemolysis when used.

#### 3.3.3. In Vivo Biocompatibility of Liposomes After Inhalation

**Figure 5:**
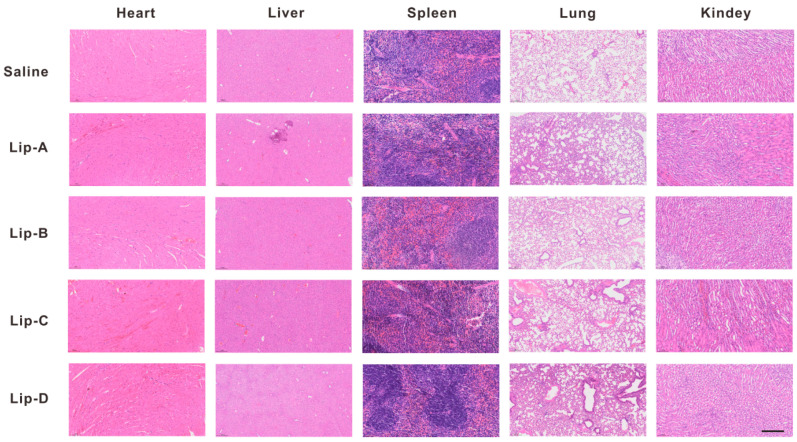
H&E staining of typical organs after inhalation of different liposomes. Scale bar, 100 μm.

In vivo biocompatibility of the liposomes after inhalation was analyzed by HE staining of typical organs. As shown in Figure 5, compared to normal mouse tissues (such as heart, liver, spleen, lungs and kidneys), no significant necrosis and inflammation was observed in the Lip-A, Lip-B, and Lip-C groups, while more obvious inflammation was observed in Lip-D group.

### 3.4. Mucus Permeability of Liposomes

**Figure 6:**
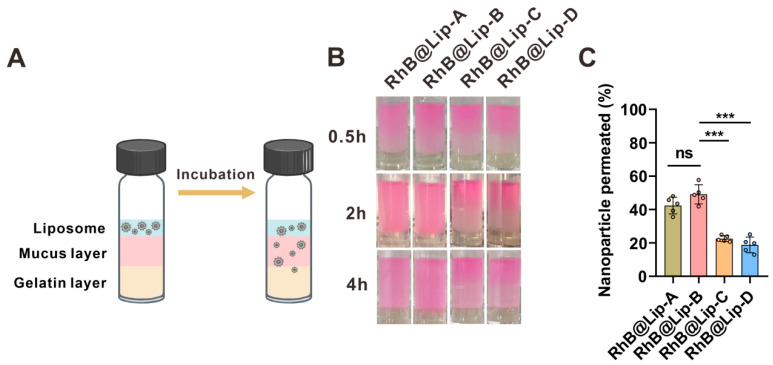
Mucus penetration of charged liposomes. (**A**) Scheme of the artificial mucus layer and the in vitro penetration test. (**B**) Typical images of the penetration of RhB@Lip (red color) into the artificial mucus layer at indicated timepoint. (**C**) The absorbance of gelatin layers after incubation with differently charged RhB@Lip at an absorbance of 552 nm (*n* = 5). Statistical significance was calculated via Student’s *t*-test. All data are expressed as mean ± SD, *** *p* < 0.001.

As shown in Figure 6, the negatively charged liposomes of the RhB@Lip-A and RhB@Lip-B groups penetrated more quickly than positively-charged liposomes, as verified by direct observation and absorbance detection of the gelatin layer. The reason might be ascribed to the stronger electronic interactions between mucus (negatively charged) [33,34] and positive liposomes, which hindered the movement of liposomes within the mucus.

### 3.5. Cellular Uptake of RhB@Lip-A and RhB@Lip-B

**Figure 7:**
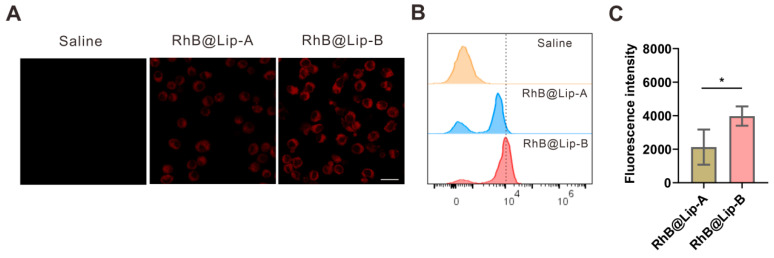
(**A**) Confocal fluorescence images of RAW247.6 cells after incubation with RhB@Lip-A and RhB@Lip-B. Scale bar 50 μm. (**B**) Cellular uptake of RhB@Lip-A and RhB@Lip-B by RAW247.6 cells analyzed by flow cytometry. (**C**) The fluorescence intensity of RhB in RAW264.7 cells (*n* = 4). All data are expressed as mean ± SD, * *p* < 0.05.

The cellular uptake of the negative liposome (Lip-A) and slightly-negative liposome (Lip-B) were evaluated. As shown in Figure 7A, the cellular uptake of RhB@Lip-B (slightly negative) by RAW264.7 cells was more obvious than that of RhB@Lip-A (negative). The phenomenon was further confirmed by data from flow cytometry. As shown in Figure 7B,C, the fluorescence signal of RhB in RAW264.7 cells was significantly higher in the RhB@Lip-B group than in RhB@Lip-A, indicating a higher potential of cellular uptake efficiency by macrophages of slightly-negative liposomes (−2.5 ± 1.1 mV) than negative-charged liposomes (−25.9 ± 2.6 mV).

Therefore, considering biocompatibility, mucus permeability and cellular uptake (related to anti-inflammation efficacy), slightly-negative liposomes (Lip-B) were more suitable as the drug carrier in this system.

### 3.6. In Vivo Anti-Inflammation Efficacy of Inhaled Drug-Loaded Liposomes

**Figure 8:**
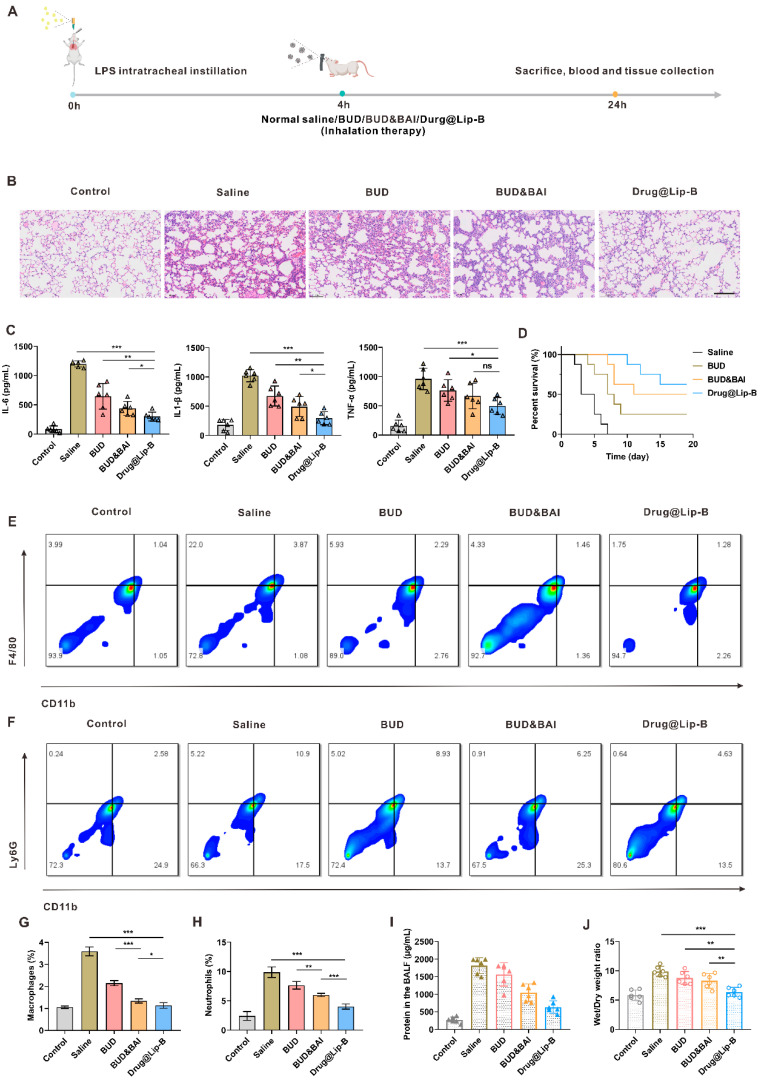
In vivo anti-inflammatory efficacy of inhaled drug-loaded liposomes in LPS-induced acute pneumonia model. (**A**) Scheme of model set-up and preparation treatment schedule. (**B**) Typical images of lung tissue after HE staining in different groups. Scale bar, 100 μm. (**C**) Serum cytokine levels of IL-6, IL-1*β*, and TNF-*α* in different groups (*n* = 6). (**D**) Survival of the mice in different treatment groups (*n* = 8). Typical flow cytometry images and percentages of macrophages in BAL fluid (**E**,**G**) (*n* = 4). Typical flow cytometry images and percentages of neutrophils in BAL fluid (**F**,**H**) (*n* = 4). (**I**) Total protein level in BAL fluid (*n* = 6). (**J**) Wet/dry weight ratio of lung tissue (*n* = 6). Statistical significance was calculated via ordinary one-way ANOVA. All data are expressed as mean ± SD, * *p* < 0.05, ** *p* < 0.01, *** *p* < 0.001.

The mice were intratracheally instilled with LPS to build up a pneumonia model, and treated by inhalation with different formulations of saline, BUD, BUD and BAI and Drug@Lip-B (Figure 8A). As shown in Figure 8B, compared with the other groups, Drug@Lip-B group showed the least lung damage and the most significant inflammation improvement, with thinning of alveolar walls and reduced inflammatory cell infiltration. Serum cytokine levels are direct indicators for evaluating anti-inflammatory efficacy; thus, the serum cytokine levels were measured to evaluate the in vivo anti-inflammatory efficacy of Drug@Lip-B administered by inhalation. Compared with other groups, the mice of Drug@Lip-B treatment group had significantly lowered levels of cytokine secretion of IL-6, TNF-*α*, and IL-1*β*, and the therapeutic efficacy was superior to that of the free BUD and BUD and BAI treatment groups (Figure 8C). Inhalation treatment with Drug@Lip-B prolonged the survival of mice with pneumonia. In detail, all mice in the saline group (treated with no drugs) died within 7 days after the inflammation model set-up, whereas 25.0% (BUD), 50.0% (BUD and BAI) and 62.5% (Drug@Lip-B) of mice survived in the indicated groups (Figure 8D). The infiltration of monocyte-derived macrophages (F4/80^+^CD11b^+^) and neutrophils (Ly6G^+^CD11b^+^) was also reduced in BALF after treatment with Drug@Lip-B, as analyzed by flow cytometry after fluorescent labelling of cells in BALF (Figure 8E–H and Figures S1 and S2). BALF total protein content is an important indicator for assessing alveolar protein exudation. As shown in Figure 8I, the total protein concentration in the Drug@Lip-B group was significantly lower than that in all other experimental groups. The lung tissue W/D ratio could be used to assess the severity of pulmonary oedema. As shown in Figure 8J, the W/D ratio also decreased the most in Drug@Lip-B-treated mice. The above data certify the superior anti-inflammation effect of the inhalation of Drug@Lip-B.

## 4. Discussion

Acute pneumonia is an acute lung infection caused by a variety of pathogenic microorganisms [35] with high morbidity and mortality. Glucocorticoids are important therapeutic drugs for the control of inflammation [36]. However, their obvious side effects [37,38], such as osteonecrosis of the femoral head and excessive suppression of the immune system, limit their clinical practice [39]. BUD and BAI are both widely used anti-inflammation drugs in China, and can exert anti-inflammatory effects through different signaling pathways [40,41]. Through evaluating the cytokine secretion of immune cells after drug treatments, our work shows that the anti-inflammatory effect of BUD and BAI combined was better than that of BUD or BAI alone (Figure 2C). The combination of BUD and BAI can improve the efficacy of glucocorticoids and reduce side effects.

The local administration of drugs by pulmonary inhalation for the treatment of pulmonary diseases has attracted considerable attention in recent years [42,43], and various nanoparticles bring new prospects for the development of pulmonary delivery systems [44,45,46]. The lung clearance and potential cytotoxicity of inhaled nanoparticles are the main factors in formulation design [10,19,47].

The particle size of inhaled liposomes has been studied thoroughly [48,49]. Some studies have shown that the mucus layer of the lung airway is composed of a sieve structure formed by mucin monomers with a sieve aperture of 200–300 nm, and particles smaller than 200 nm are more able to penetrate the mucus layer [50,51]. Thus, liposomes with particle sizes 100~200 nm are suitable for inhalation. Therefore, in this work the particle size were controlled between 100~120 nm (Table 1).

The surface charge of nanoparticles also significantly influences the drug efficacy of an inhalation delivery system. In detail, the airway mucus layer consists primarily of negatively charged mucins [33], and mucociliary clearance is the main defense mechanism of the upper conduction airway against foreign bodies. Thus, if the particles are trapped by the mucus layer, they will be rapidly cleared via the oscillating mucociliary clearance [26,27]. From this aspect, the inhaled nanoparticles should minimize undesirable interactions with mucin and avoid clearance by mucus trapping before reaching the target cells. In this work, we prepared liposomes with four different surface charges, Lip-A (−25.9 mV, negative), Lip-B (−2.5 mV, slightly negative), Lip-C (2.6 mV, slightly positive) and Lip-D (21.5 mV, positive). In vitro mucus penetration results showed the negatively charged liposomes were of significantly superior penetration capability compared with positively charged liposomes. Meanwhile, negatively charged liposomes also exhibited advantages in formulation stability (Table S2), cytotoxicity (Figure 3) and blood compatibility (Figure 4).

Besides mucus penetration, cellular uptake is also important for drugs. The data showed that the cellular uptake of slightly negative-charged liposomes (−2.5 mV) was better than that of heavily negative-charged liposomes (−25.9 mV) (Figure 7), even though they exhibited similar mucus penetration abilities (Figure 6) and both presented enhanced pulmonary retention and low systemic exposure [52]. Thus, the slightly negative-charged liposomes could achieve a proper balance of good mucus penetration and cellular uptake, and improve the drug efficacy of lung inhalation formulations for treatment of pneumonia.

## 5. Conclusions

The anti-inflammatory effect of BUD and BAI combined was better than that of BUD or BAI alone. Slightly negative-charged liposomes could achieve a proper balance of good mucus penetration and cellular uptake, and were a proper delivery vehicle for lung inhalation delivery systems, which could significantly improve the anti-inflammation efficacy of loaded drugs.

## Figures and Tables

**Table 1:** Parameters of drug-loaded liposomes.

	Particle Size (nm)	PDI	Zeta Potential (mV)	EE (%)		DL (%)	
				BUD	BAI	BUD	BAI
Drug@Lip-A (negative)	117.1 ± 1.2	0.39 ± 0.04	−25.9 ± 2.6	73.9 ± 9.1	70.0 ± 15.8	3.4 ± 3.0	4.1 ± 2.5
Drug@Lip-B (slightly negative)	109.7 ± 1.8	0.26 ± 0.01	−2.5 ± 1.1	73.2 ± 5.3	73.0 ± 4.6	4.2 ± 2.0	6.2 ± 0.7
Drug@Lip-C (slightly positive)	120.9 ± 1.4	0.28 ± 0.01	2.6 ± 0.4	68.9 ± 10.7	68.4 ± 21.6	1.9 ± 0.9	1.9 ± 1.1
Drug@Lip-D (positive)	113.5 ± 1.6	0.33 ± 0.02	21.5 ± 1.5	71.0 ± 7.7	64.9 ± 15.2	3.2 ± 1.7	4.6 ± 3.2

## Data Availability

The original contributions presented in this study are included in the article. Further inquiries can be directed to the corresponding author.

## Supplementary Materials

The following supporting information can be downloaded at: https://www.mdpi.com/article/10.3390/pharmaceutics17030329/s1, Table S1. Combination ratio and index of budesonide (BUD) and baicalin (BAI). Table S2. The size change of Drug@Lip after storing in PBS at 4 °C (*n* = 3). Data are shown as mean ± SD. Figure S1. Flow cytometry gating strategy. (A) Blank cells without fluorescence labeling. (B) Flow cytometry gating strategy for macrophages (CD11b^+^F4/80^+^ cells). Figure S2. Flow cytometry gating strategy. (A) Blank cells without fluorescence labeling. (B) Flow cytometry gating strategy for neutrophils (CD11b^+^Ly6G^+^ cells).

## References

[1] Zheng M.L., Zhu W., Gao F., Zhuo Y., Zheng M., Wu G.H., Feng C.L. (2024). Novel inhalation therapy in pulmonary fibrosis: Principles, applications and prospects. J. Nanobiotechnol..

[2] Izquierdo-Condoy J.S., Salazar-Santoliva C., Salazar-Duque D., Palacio-Dávila Y.D., Hernández-Londoño J.M., Orozco-Gonzalez R., Rodríguez-Sánchez M.S., Marín-Bedoya V., Loaiza-Guevara V. (2024). Challenges and opportunities in COPD management in Latin America: A review of inhalation therapies and advanced drug delivery systems. Pharmaceutics.

[3] Zhang M.J., Lu H.Y., Xie L.K., Liu X.L., Cun D.M., Yang M.S. (2023). Inhaled RNA drugs to treat lung diseases: Disease-related cells and nano-bio interactions. Adv. Drug Deliv. Rev..

[4] Deng X., Yang Y., Gan L., Duan X., Wang X., Zhang J., Wang A., Zhang A., Yuan Z., Chen D. (2025). Engineering lipid nanoparticles to enhance intracellular delivery of transforming growth factor-beta siRNA (siTGF-β1) via inhalation for improving pulmonary fibrosis post-bleomycin challenge. Pharmaceutics.

[5] Liu C., Xi L., Liu Y., Mak J.C.W., Mao S., Wang Z., Zheng Y. (2023). An inhalable hybrid biomimetic nanoplatform for sequential drug release and remodeling lung immune homeostasis in acute lung injury treatment. ACS Nano.

[6] Anderson C.F., Grimmett M.E., Domalewski C.J., Cui H. (2020). Inhalable nanotherapeutics to improve treatment efficacy for common lung diseases. Wiley Interdiscip. Rev. Nanomed. Nanobiotechnol..

[7] Wang B., Wang L., Yang Q., Zhang Y.M., Qinglai T., Yang X.M., Xiao Z., Lei L.J., Li S.S. (2024). Pulmonary inhalation for disease treatment: Basic research and clinical translations. Mater. Today Bio.

[8] Raviv S.A., Alyan M., Egorov E., Zano A., Harush M.Y., Pieters C., Korach-Rechtman H., Saadya A., Kaneti G., Nudelman I. (2022). Lung targeted liposomes for treating ARDS. J. Control. Release.

[9] He S.Q., Gui J.J., Xiong K., Chen M.W., Gao H.L., Fu Y. (2022). A roadmap to pulmonary delivery strategies for the treatment of infectious lung diseases. J. Nanobiotechnol..

[10] Yue P.F., Zhou W.C., Huang G.T., Lei F.F., Chen Y.C., Ma Z.L., Chen L.R., Yang M. (2022). Nanocrystals based pulmonary inhalation delivery system: Advance and challenge. Drug Deliv..

[11] Ge C.L., Yang J.D., Duan S.Z., Liu Y., Meng F.H., Yin L.C. (2020). Fluorinated α-Helical polypeptides synchronize mucus permeation and cell penetration toward highly efficient pulmonary siRNA delivery against acute lung injury. Nano Lett..

[12] Hua S., Hu H., Liu J., Lu F., Yu R., Zhang X., Sun H., Wang Z., Li Y., Xia J. (2024). A mucous permeable local delivery strategy based on manganese-enhanced bacterial. ACS Nano.

[13] Xu Z., Bera H., Wang H., Wang J., Cun D., Feng Y., Yang M. (2023). Inhalable ciprofloxacin/polymyxin B dry powders in respiratory infection therapy. AMM.

[14] Dattani S., Li X., Lampa C., Barriscale A., Damadzadeh B., Lechuga-Ballesteros D., Jasti B.R. (2025). Development of spray-dried micelles, liposomes, and solid lipid nanoparticles for enhanced stability. Pharmaceutics.

[15] Lazo R.E.L., Mengarda M., Almeida S.L., Caldonazo A., Espinoza J.T., Murakami F.S. (2022). Advanced formulations and nanotechnology-based approaches for pulmonary delivery of sildenafil: A scoping review. J. Control. Release.

[16] Lee W.H., Loo C.Y., Traini D., Young P.M. (2015). Nano- and micro-based inhaled drug delivery systems for targeting alveolar macrophages. Expert Opin. Drug Deliv..

[17] Kruijf W.d., Ehrhardt C. (2017). Inhalation delivery of complex drugs—The next steps. Curr. Opin. Pharmacol..

[18] Kansız S., Elçin Y.M. (2023). Advanced liposome and polymersome-based drug delivery systems: Considerations for physicochemical properties, targeting strategies and stimuli-sensitive approaches. Adv. Colloid Interface Sci..

[19] Bassetti M., Vena A., Russo A., Peghin M. (2020). Inhaled liposomal antimicrobial delivery in lung infections. Drugs.

[20] Shirley M. (2019). Amikacin liposome inhalation suspension: A review in mycobacterium avium complex lung disease. Drugs.

[21] Peng S.Y., Wang W.H., Zhang R., Wu C.B., Pan X., Huang Z.W. (2024). Nano-formulations for pulmonary delivery: Past, present, and future perspectives. Pharmaceutics.

[22] Jang M., Yeom K., Han J., Fagan E., Park J.H. (2024). Inhalable mRNA nanoparticle with enhanced nebulization stability and pulmonary microenvironment infiltration. ACS Nano.

[23] Neary M.T., Mulder L.M., Kowalski P.S., MacLoughlin R., Crean A.M., Ryan K.B. (2024). Nebulised delivery of RNA formulations to the lungs: From aerosol to cytosol. J. Control. Release.

[24] Kassaee S.N., Ayoko G.A., Richard D., Wang T., Islam N. (2024). Inhaled ivermectin-loaded lipid polymer hybrid nanoparticles: Development and characterization. Pharmaceutics.

[25] Wang Y., Zhang J., Liu Y., Yue X., Han K., Kong Z.C., Dong Y.M., Yang Z.M., Fu Z.P., Tang C.W. (2024). Realveolarization with inhalable mucus-penetrating lipid nanoparticles for the treatment of pulmonary fibrosis in mice. Sci. Adv..

[26] Khutoryanskiy V.V. (2017). Beyond PEGylation: Alternative surface-modification of nanoparticles with mucus-inert biomaterials. Adv. Drug Deliv. Rev..

[27] Shan W., Zhu X., Liu M., Li L., Zhong J., Sun W., Zhang Z., Huang Y. (2015). Overcoming the diffusion barrier of mucus and absorption barrier of epithelium by self-assembled nanoparticles for oral delivery of insulin. ACS Nano.

[28] Wang H., Zuo Z., Du J., Wang Y., Sun R., Cao Z., Ye X., Wang J., Leong K., Wang J. (2016). Surface charge critically affects tumor penetration and therapeutic efficacy of cancer nanomedicines. Nano Today.

[29] Rane S.S., Choi P. (2005). Polydispersity index: How accurately does it measure the breadth of the molecular weight distribution? (Note). Chem. Mater..

[30] Danaei M., Dehghankhold M., Ataei S., Hasanzadeh Davarani F., Javanmard R., Dokhani A., Khorasani S., Mozafari M.R. (2018). Impact of particle size and polydispersity index on the clinical applications of lipidic nanocarrier systems. Pharmaceutics.

[31] Wu J., Zhai T., Sun J., Yu Q., Feng Y., Li R., Wang H., Ouyang Q., Yang T., Zhan Q. (2022). Mucus-permeable polymyxin B-hyaluronic acid/ poly (lactic-co-glycolic acid) nanoparticle platform for the nebulized treatment of lung infections. J. Colloid Interface Sci..

[32] Wang Y., Ding Q., Ma G., Zhang Z., Wang J., Lu C., Xiang C., Qian K., Zheng J., Shan Y. (2024). Mucus-penetrable biomimetic nanoantibiotics for pathogen-induced pneumonia treatment. ACS Nano.

[33] Yan X., Sha X.Y. (2023). Nanoparticle-mediated strategies for enhanced drug penetration and retention in the airway mucosa. Pharmaceutics.

[34] Yang M.Y., Han M.M., Tang L., Bi Y.Y., Li X.N., Jeong J.H., Wang Y., Jiang H.L. (2024). Dual barrier-penetrating inhaled nanoparticles for enhanced Idiopathic pulmonary fibrosis therapy. Adv. Funct. Mater..

[35] Quinton L.J., Walkey A.J., Mizgerd J.P. (2018). Integrative physiology of pneumonia. Physiol. Rev..

[36] Altube M.J., Perez N., Romero E.L., Morilla M.J., Higa L.H., Perez A.P. (2023). Inhaled lipid nanocarriers for pulmonary delivery of glucocorticoids: Previous strategies, recent advances and key factors description. Int. J. Pharm..

[37] Pirracchio R., Venkatesh B., Legrand M. (2024). Low-dose corticosteroids for critically Ill adults with severe pulmonary. JAMA.

[38] Dallal Bashi Y.H., Mairs R., Murtadha R., Kett V. (2025). Pulmonary delivery of antibiotics to the lungs: Current state and future prospects. Pharmaceutics.

[39] Brustad N., Buchvald F., Jensen S.K., Kyvsgaard J.N., Vahman N., Thorsen J., Schoos A.-M.M., Nygaard U., Vissing N., Stokholm J. (2025). Burden of infections in early life and risk of infections and systemic antibiotics use in childhood. JAMA Netw. Open.

[40] Yan Y., Wang P., Li R., Sun Y., Zhang R., Huo C., Xing J., Dong Y. (2019). Synthesis of budesonide conjugates and their anti-inflammatory effects: A preliminary study. Drug Des. Dev. Ther..

[41] Zhao Q., Chen X.-Y., Martin C. (2016). *Scutellaria baicalensis*, the golden herb from the garden of Chinese medicinal plants. Sci. Bull..

[42] Backer L.D., Cerrada A., Pérez-Gil J., Smedt S.C.D., Raemdonck K. (2015). Bio-inspired materials in drug delivery: Exploring the role of pulmonary surfactant in siRNA inhalation therapy. J. Control. Release.

[43] Sheng G., Tian N., Duan H., Sun Z., Chu H. (2022). Advances in therapeutic nanodrug delivery systems for infectious lung diseases: A review. AMM.

[44] García-Fernández A., Sancenón F., Martínez-Máñez R. (2021). Mesoporous silica nanoparticles for pulmonary drug delivery. Adv. Drug Deliv. Rev..

[45] Liao R., Sun Z.C., Wang L., Xian C., Lin R., Zhuo G., Wang H., Fang Y., Liu Y., Yang R. (2024). Inhalable and bioactive lipid-nanomedicine based on bergapten for targeted acute lung injury therapy via orchestrating macrophage polarization. Bioact. Mater..

[46] Fernández-García R., Fraguas-Sánchez A.I. (2024). Nanomedicines for pulmonary drug delivery: Overcoming barriers in the treatment of respiratory infections and lung cancer. Pharmaceutics.

[47] Fu F., Wang W., Wu L., Wang W., Huang Z., Huang Y., Wu C., Pan X. (2023). Inhalable biomineralized liposomes for cyclic Ca2+-burst-centered endoplasmic reticulum stress enhanced lung cancer ferroptosis therapy. ACS Nano.

[48] Scialabba C., Craparo E.F., Cabibbo M., Drago S.E., Cavallaro G. (2024). Exploiting inhalable microparticles incorporating hybrid polymer-lipid nanoparticles loaded with Iloprost manages lung hyper-inflammation. Int. J. Pharm..

[49] Scialabba C., Craparo E.F., Bonsignore S., Cabibbo M., Cavallaro G. (2024). Lipid–polymer hybrid nanoparticles in microparticle-based powder: Evaluating the potential of methylprednisolone delivery for future lung disease treatment via inhalation. Pharmaceutics.

[50] Behnen M., Möller S., Brozek A., Klinger M., Laskay T. (2017). Extracellular acidification inhibits the ROS-dependent formation of neutrophil. Front. Immunol..

[51] Asmawi A.A., Salim N., Abdulmalek E., Abdul Rahman M.B. (2023). Size-controlled preparation of docetaxel- and curcumin-loaded nanoemulsions for potential pulmonary delivery. Pharmaceutics.

[52] Zhao J., Qin L., Song R., Su J., Yuan Y., Zhang X., Mao S. (2022). Elucidating inhaled liposome surface charge on its interaction with biological barriers in the lung. Eur. J. Pharm. Biopharm..

