# Supplementary material for: Effects of Surface Charge of Inhaled Liposomes on Drug Efficacy and Biocompatibility
Source: Pharmaceutics. 2025 Mar 3;17(3):329. doi: 10.3390/pharmaceutics17030329 (PMC11945262; doi:10.3390/pharmaceutics17030329)
Supplement: Supplementary file 1 [file pharmaceutics-17-00329-s001.zip › pharmaceutics-3497639-supplementary.docx]

Supplementary Information

Effects of Surface Charge of Inhaled Liposomes on Drug
Efficacy and Biocompatibility

Jinniu Zhang, Yun Huang, Wenhao Shen, Yixing Zeng, Yingjing Miao, Nianping Feng * and Tianyuan Ci *

School of Pharmacy, Shanghai University of Traditional Chinese Medicine, Shanghai, 201203, China

***** Correspondence: Corresponding authors: tianyuanci@shutcm.edu.cn (T.C.); npfeng@shutcm.edu.cn (N.F.)

This PDF file includes:

Table S1. Combination ratio and index of budesonide (BUD) and baicalin (BAI).

Table S2. The size change of Drug@Lip after storing in PBS at 4℃ (*n* = 3). Data are shown as mean ± SD.

Figure S1. Flow cytometry gating strategy. (A) Blank cells without fluorescence labeling. (B) Flow cytometry gating strategy for macrophages (CD11b^+^F4/80^+^ cells).

Figure S2. Flow cytometry gating strategy. (A) Blank cells without fluorescence labeling. (B) Flow cytometry gating strategy for neutrophils (CD11b^+^Ly6G^+^ cells).

**Table S1.** Combination ratio and index of BUD and BAI.

| Drug | Concentration of BUD [μM] | Concentration of BAI [μM] | Mass Ratio | CI |
| --- | --- | --- | --- | --- |
| IC_50, BUD_ | 2.13 |  |  |  |
| 95% IC_50, BUD_ :  5% IC_50, BAI_ | 2.02 | 0.89 | 2.00:1 | 0.15 |
| 85% IC_50, BUD_ : 15% IC_50, BAI_ | 1.81 | 2.67 | 1:1.68 | 0.14 |
| 60% IC_50, BUD_ : 40% IC_50, BAI_ | 1.28 | 7.13 | 1:6.34 | 2.80 |
| 50% IC_50, BUD_ : 50% IC_50, BAI_ | 1.07 | 8.91 | 1:9.47 | 1.74 |
| 40% IC_50, BUD_ : 60% IC_50, BAI_ | 0.85 | 10.70 | 1:14.32 | 0.81 |
| 15% IC_50, BUD_ : 85% IC_50, BAI_ | 0.32 | 15.15 | 1:53.84 | 0.74 |
| 5% IC_50, BUD_ :  95% IC_50, BAI_ | 0.11 | 16.93 | 1:175.04 | 0.29 |
| IC_50, BAI_ |  | 17.82 |  |  |

**Table S2.** Particle size of Drug@Lip after storing in PBS at 4℃ (*n* = 3).

| Sample | Particle Size of Liposomes (nm) | | | |
| --- | --- | --- | --- | --- |
|  | **1d** | **7d** | **14d** | **25d** |
| Drug@Lip-A  (negative) | 117.13 ± 1.21 | 120.07 ± 1.31 | 129.2 ± 3.40 | 137.87 ± 2.75 |
| Drug@Lip-B  (slightly negative) | 109.67 ± 1.76 | 105.40 ± 0.66 | 117.13 ± 0.50 | 126.63 ± 2.02 |
| Drug@Lip-C  (slightly positive) | 120.90 ± 1.39 | 119.23 ± 2.05 | 128.63 ± 1.29 | 130.33 ± 3.51 |
| Drug@Lip-D  (positive) | 113.53 ± 1.63 | 143.00 ± 1.73 | 143.57 ± 2.55 | 146.23 ± 6.33 |


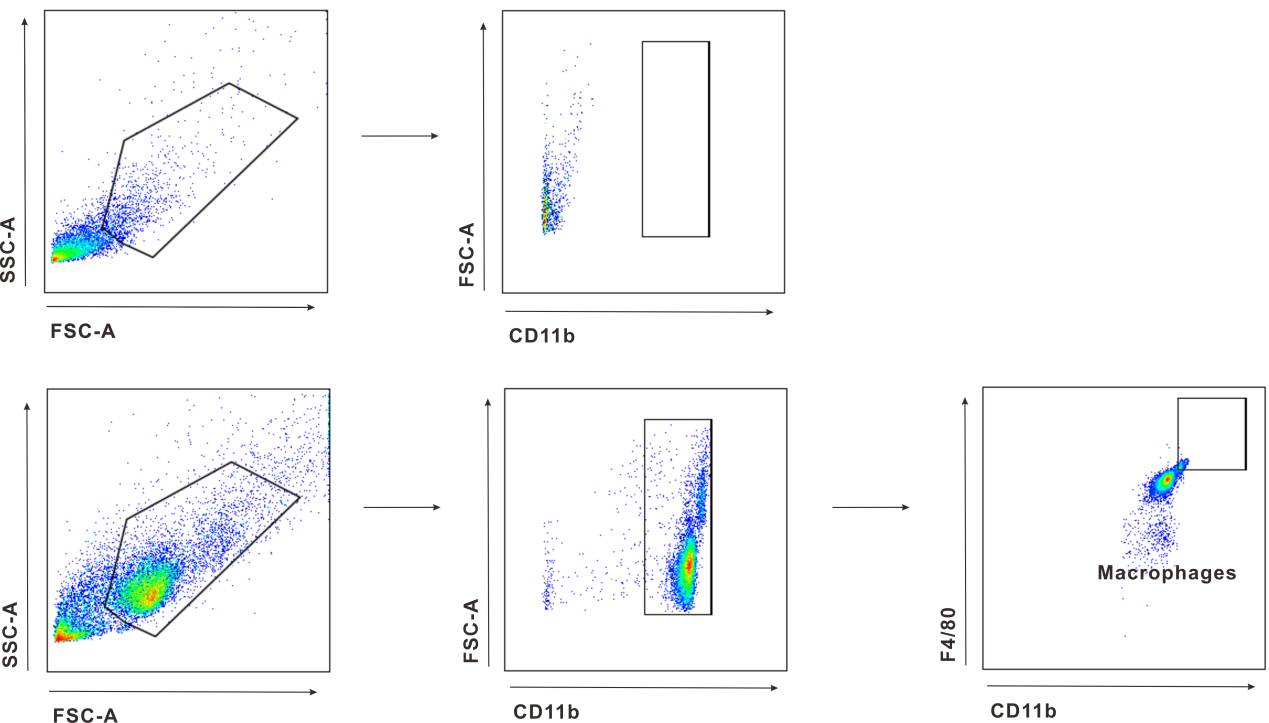


**Figure S1.** Flow cytometry gating strategy. (A) Blank cells without fluorescence labeling. (B) Flow cytometry gating strategy for macrophages (CD11b^+^F4/80^+^ cells).


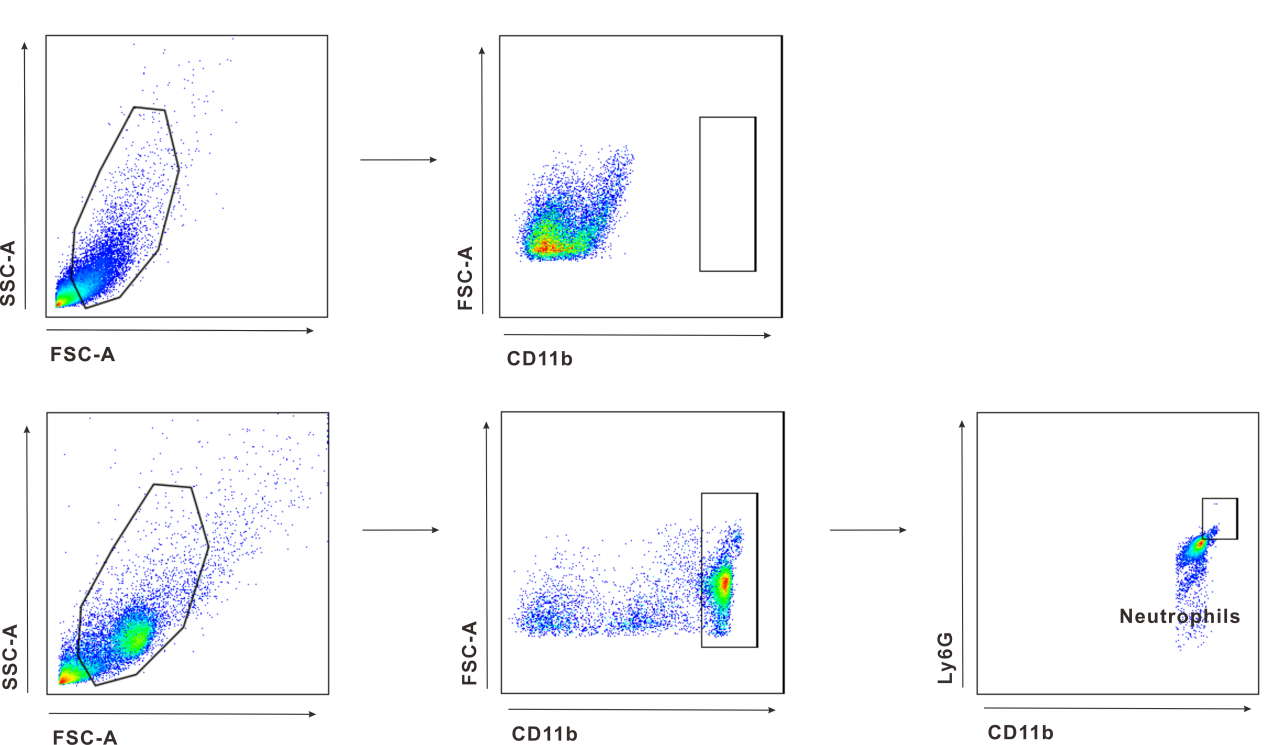


**Figure S2.** Flow cytometry gating strategy. (A) Blank cells without fluorescence labeling. (B) Flow cytometry gating strategy for neutrophils (CD11b^+^Ly6G^+^ cells).
